# Military Camp Sanitation

**Published:** 1898-10

**Authors:** 


Monthly Review of Medicine and Surgery.

*EDITORS:*

THOMAS LOTHROP, M. D. - - WM. WARREN POTTER, M. D.

All communications, whether of a literary or business nature, should be addressed to the managing editor: 284 FRANKLIN STREET, BUFFALO, N. Y.

VOL. XXXVIII. OCTOBER, 1898. No. 3

### MILITARY CAMP SANITATION.

DURING the progress of the war with Spain, now so happily and victoriously concluded, it has been the custom of the JOURNAL to speak editorially in each number of some phase of the army medical service, and especially to uphold the Surgeon-General and his aides and subordinates in the trying duty that was so suddenly thrust upon them. Now, however, since an investigation into the management of the war department has been ordered by the President, we shall refrain from further comment on this subject until the commission shall present its report. It would be manifestly indelicate to offer further criticism, pro or con, during the progress of the investigation.

Military medical affairs are, however, still so much in evidence that it is yet pertinent to discuss them from the viewpoint of the sanitarian, whose office is quite as important in military as in civil life. Let us, therefore, turn our attention for a moment to the consideration of the problem of camp sanitation, which, in view of experience in the late war, invites the most serious consideration.

Heretofore the genius of our republican institutions has decreed against a large standing army, hence, in emergencies, we are compelled suddenly to raise large quotas of volunteers and put them promptly into the field. This of necessity means hardship, exposure, sickness and death in a degree out of proportion to an equal number of our male fellow-citizens of adult age in civil life. These new soldiers are, for the most part, ignorant as to the simplest laws of preventive medicine, and especially as to the fact that the most dangerous enemies of the soldier are the water-borne diseases. It is difficult to make a thirsty soldier refrain from drinking the most available water when it does not present to the eye an abhorrent appearance; and even under extreme conditions he will not be restrained from gratifying his thirst at the most repellant stream. If diseases of the intestinal tract, that are by far the most menacing to the welfare of the soldier, are to be controlled, it becomes the government to look well to the water supply of its camps of instruction. Here, in our view, is the place of all others where the laws of prevention can be applied with the expectation of a large measure of success. When the army is on the march in an active campaign, camps must be chosen where they will, where the exigency of military necessity insists. In such event it matters little where the bivouac or temporary camp is pitched, since the stay is of short duration and everything must bend to the strategic demands. But with the camps of instruction, or permanent camps of occupation, the conditions are totally different. Here everything else should yield to the question of sanitation—to the problem of how best to preserve the health of the soldier and harden him for his future work.

First of all comes the question of good pure water in ample supply. All other considerations may be placed in the secondary list. Let the selection be made with reference to good porous soil, drainage facilities that cannot be challenged, and without a well or spring within miles. If these are near by they are almost certain, in spite of vigorous precautions, to become contaminated. The water supply should be well chosen as to quality and quantity, strongly guarded at its fountain-head, and then piped to the camp. In this way only can the water-borne diseases be practically reduced to the minimum. To partake of water from any other source should be attended with penalty.

We shall limit our observations at this time to this one point connected with military camp sanitation, believing it to be paramount to all others. With an ample, pure water supply intestinal diseases, such as diarrheas and typhoid fever, ought to be reduced to a comparatively insignificant minimum. With these diseases abated the others may receive attention as they arise. The question of expense in the preparation of camps of instruction or occupation need not enter into the account. The most expensive element the war department has to deal with is a sick soldier. He is not only a great care, but he hinders the progress of the army, and his presence, multiplied by hundreds and thousands, may mean defeat where victory would otherwise assuredly result. Camp sites, such as are under discussion, should be located by a commission appointed by the surgeongeneral and their healthfulness assured by constant rigid inspection at the hands of the most competent men for the service at command.

---

LAST month we referred to the number of casualties occurring in the war with Spain. These reports are still subject to revision. A late examination of the official records of the War Department, as far as completed, show that there were 33 officers and 231 enlisted men of the army, 264 in all, killed in battle. These casualties include all the lives lost by the army in the battles in the Philippines, as well as in those in Cuba and Porto Rico. The percentage of officers killed is strikingly large, and is said to be unprecedented in the battles of the world. The contrast is especially striking in the case of the battle of Omdurman, where, although the loss of life was heavy, the list of killed included only one officer of the British army.

---

IF LIEUT.-COL. NICHOLAS SENN, U. S. Volunteers, late chief of the operating staff of the armies in the field, sometimes erroneously called assistant-surgeon general, had written less and worked more, it would have been better for his own reputation at least, if not for the service itself. Not that Professor Senn has not worked hard, for he is capable of great endurance, but he owed it to himself and the government to devote *all* his time to the soldiers. The records and notes, such as are for public use, had better be written up after the war, when there is time for assimilation and digestion and when calm judgment comes to assist in sorting, retaining such as are of value and rejecting others of no consequence. When an officer of the medical staff writes reflectingly upon his commanding officers, he may at least be regarded with suspicion, and whatever he writes must of necessity be taken with a grain of sand. If any of the commanders must be tried at the bar of public opinion, let it be reserved for the yellow newspapers and the idle home-stayers to bring the indictment, and not for the staff, least of all the medical staff.

---

THE death of Dr. George W. Lindheim, of the 8th New York Regiment of Infantry, which occurred September 16, 1898, presents one of the saddest pictures of the war. Charged with the duty of escorting 260 sick soldiers from Chickamauga to New York, his journey was constantly set with difficulties and dangers. At Cleveland it is stated that some of the citizens, physicians among the number, boarded the train and demanded that a number of the sick be left at the hospitals in that city. It is even alleged that threats were made in case the demand was not acceded to. But the faithful doctor carried out his orders to deliver the train load of sick in New York and declined the proposal of the Cleveland people.

At Buffalo some well-meaning, though misguided philanthropic ladies insisted upon serving the fever-stricken soldiers with ham sandwiches, and the like, all of which Dr. Lindheim resisted, as was his duty. Nevertheless, even some very good newspapers—one, we are sorry to admit, printed in Buffalo—roundly abused Dr. Lindheim for his action, and some ladies, we have heard, spoke of him in terms of which they ought to be ashamed.

The sequel is that Dr. Lindheim, after delivering his charge in New York to the proper hospitals, himself fell sick of the fever, for which the ladies of Buffalo prescribed ham sandwiches, and after a few days of struggle finally succumbed to the disease, a victim to the unkind, not to say, wicked and malignant criticism of the newspapers and the public. In his delirium he defended his soldiers against the assaults of the newspapers and the ham sandwich brigade:

"Let me get up!" he raved. "Two hundred and sixty of them. Think of the boys—so much responsibility—I must get up."

Then the three nurses constantly by his side did all that could be done to quiet him. Again the sufferer called out:

"Oh, why do they judge me unheard? I'm doing all I can; I'm right; I know I'm right. I'll bring them all through."

And so this high-minded patriotic soldier breathed out his life, a victim to the awful freedom of the press and the but little less awful freedom of speech.

---

THE Secretary of War has issued an order requiring that a record of the physical condition of every volunteer soldier at the date of his discharge from the military service of the United States shall be made and preserved among the permanent records of the war department. This is an important matter both for the soldier and the government, and will have great bearing on the adjustment of claims for pensions. About 500 such claims have already been filed with the commissioner of pensions, and more are likely to follow.

This order of the Secretary of War prescribes that the record shall consist, first, of the soldier's own declaration as to his physical condition and of any reasons why, in his opinion, it has become impaired as a result of his military service; second, of the statement and certificate of his company or other immediate commander respecting the case; and, third, of the certificate of a surgeon who is to make a thorough physical examination. If a physical disability is claimed which the surgeon cannot find, or which, being found, did not in his opinion originate in the service in line of duty, the soldier will not be discharged until after he shall have been examined by a board of three other medical officers, who shall make a full report of the case.

If this order is carried out to completeness it will greatly facilitate the prompt adjustment of valid claims for pension, as well as protect the government against fraud.

---

COL. C. C. BYRNE, chief medical officer, Department of the East, early in September inspected the sanitary condition of Camp Black. The result was that he found everything in splendid shape in the camp itself and in its water supply. Failing at first to discover the source of typhoid fever, then prevailing to some extent, he sought elsewhere for it. During the course of his inquiry he wanted to know the kind of wells used by the farmers within a few miles of the camp, along the roads the soldiers were in the habit of wandering while out on leave. This brought to light the fact that a good portion of the wells were from fifteen to twenty feet deep, and were loosely built. Water is plentiful at from fifteen to twenty feet under ground in this neighborhood, and a well is not often deeper.

One of the surgeons then related an instance of water being carried into camp from a well that could not help being polluted. A soldier returning from leave of absence had two full canteens, and was stopped to show what was in them. It was water, and the man explained that he and some of the others liked the water from a certain well, and were in the habit of getting a supply daily, one man in the combination going out every day.

The surgeon who overheard this obtained permission for the soldier to show him this well. When reached it proved to be less than twenty feet deep, and within twelve feet of it was a privy vault. The well was of the loose-built kind, and the walls of the vault were simply rough stone set together without cement, so that the water in the vault might soak away into the earth. The side of the vault was within twelve feet of the well, and the doctor said a steady stream must run from the vault to the well. This was bad enough, but on inquiring at the house he found in it a woman just recovering from a severe illness, which the doctor said he more than suspected was typhoid in a light form.

Here, then, is the explanation, so far as Camp Black is concerned, as to the source of typhoid fever, and, we doubt not, if an equally careful inquiry were made, its source at other camps would be found of similar origin.

---

THE annual report of the commissioner of pensions for the year ending June 30, 1898, discloses some peculiarities in regard to ratings for disability found by examining boards, that occasion complaint among the pensioners. It is an evil very difficult to remedy, since the question of personal judgment enters into the subject, and this of necessity is very variable. By way of illustrating these peculiarities and difficulties the report says :

> The practice requires that medical examiners shall make a diagnosis and a complete and accurate pen picture of the disability or disabilities of the claimant. They receive a small fee for each examination and detailed report, and such examinations often no doubt are hurried and superficial. Therefore, many of the examinations are not made. As an illustration, it is stated that the bureau found it necessary to cause a test examination to be made of a pensioner. He was sent before four medical boards, three members each, all present. All of these boards were within the classified service. Each examined the pensioner under the same instructions.
>
> Each board made a careful examination and reported the results of their investigations with the usual care, describing the pensioner's disabilities from their standpoint. Each board found unanimously ; no minority report. The results, summed up briefly, were : One board could find no ratable disability ; another found a ratable disability and estimated it at $8 per month ; another board found disability, carefully described it, and rated it at $17 a month, while the other board made equally as careful examination and estimated the claimant's disabilities as third grade—$24 a month. Same man, same conditions, same instructions, and all within forty-eight hours. These physicians are, each and all, I am informed, skilled in their practice, in every way reputable practitioners.

One of the evils in the matter of examination it seems to us lies in disclosing to attorneys and other inquirers for the claimant, the report of the examining surgeons. We see no reason why this should not be treated as confidential, just as are the ordinary relations between physician and patient. If this were so regarded we think the department would get better service.

